# Evaluating the toxicity profile of combination immune checkpoint inhibitors: a disproportionality analysis of real-world adverse events from the FDA Adverse Event Reporting System for tremelimumab, durvalumab, ipilimumab, and nivolumab

**DOI:** 10.3389/fimmu.2025.1631273

**Published:** 2025-09-01

**Authors:** Zhuoyang Li, Yuxuan Xie, Tianhong Wang, Yuwei Liu, Yining Tian, Yusi Hua

**Affiliations:** ^1^ School of Medicine, Wuhan University of Science and Technology, Wuhan, China; ^2^ Tianyou Hospital, Wuhan University of Science and Technology, Wuhan, China; ^3^ Department of Anesthesiology, West China Hospital, Sichuan University, Chengdu, China; ^4^ The Department of Clinical Research, West China Hospital, Sichuan University, Chengdu, China

**Keywords:** tremelimumab, durvalumab, ipilimumab, nivolumab, immune checkpoint inhibitor, FAERS, adverse event

## Abstract

**Background:**

As one of the therapeutic modalities for treating tumors, immune checkpoint inhibitors (ICIs) have gained widespread application in clinical practice, including non-small cell lung cancer, melanoma, head and neck squamous cell carcinoma, hepatocellular carcinoma, and other types of cancers. However, the safety profile of combining ICIs remains inadequately understood, which poses limitations on the clinical utilization of this novel class of medications. To investigate the toxicity spectrum associated with combination immunotherapy, we conducted an extensive data mining and analysis of the US Food and Drug Administration Adverse Event Reporting System (FAERS) database.

**Methods:**

By mining adverse event (AE) reports from the FAERS database covering the period from the first quarter of 2011 through the second quarter of 2024, baseline data were analyzed using Cramer’s V coefficient and p-value. Subsequently, two methods, the reporting odds ratio (ROR) and the Bayesian confidence propagation neural network, were employed to detect AE signals for single immune checkpoint inhibitors (sICIs) and dual immunotherapy group (tremelimumab plus durvalumab and ipilimumab plus nivolumab, DIG).

**Results:**

A total of 55,052 patients and 118,001 AEs were selected. The DIG exhibited a higher incidence of AE signals across 14 distinct system organ class level. Moreover, DIG exhibited higher positive signal intensity compared to sICIs in the following preferred terms: myocarditis [ROR 2.221, 95% confidence interval lower limit of information component (IC_025_) 0.486], immune-mediated myocarditis (ROR 2.922, IC_025_ 0.610), adrenal insufficiency (ROR 2.503, IC_025_ 0.602), hyperthyroidism (ROR 1.872, IC_025_ 0.305), thyroiditis (ROR 2.669, IC_025_ 0.546), immune-mediated enterocolitis (ROR 3.948, IC_025_ 0.937), pyrexia (ROR 1.570, IC_025_ 0.290), hepatic function abnormality (ROR 2.582, IC_025_ 0.591), hepatitis (ROR 2.705, IC_025_ 0.637), liver disorder (ROR 2.718, IC_025_ 0.646), immune-mediated hepatitis (ROR 5.504, IC_025_ 0.994), immune-mediated liver disorder (ROR 5.322, IC_025_ 0.966), cytokine release syndrome (ROR 7.650, IC_025_ 1.103), autoimmune diseases (ROR 1.754, IC_025_ 0.275), sepsis (ROR 1.414, IC_025_ 0.062), diabetic ketoacidosis (ROR 2.294, IC_025_ 0.472), type 1 diabetes mellitus (ROR 2.421, IC_025_ 0.508), arthritis (ROR 1.562, IC_025_ 0.113), myositis (ROR 2.204, IC_025_ 0.412), and acute kidney injury (ROR 1.708, IC_025_ 0.264).

**Conclusions:**

Our findings indicate that the AEs associated with dual ICI predominantly originate from immune-related AEs, including myotoxicity, endocrine toxicity, and hepatotoxicity. Notably, cytokine release syndrome, a rarely reported AE with a strongly positive signal, warrants particular attention in clinical decision-making.

## Introduction

As a prominent focus in tumor therapy, immune checkpoint inhibitors (ICIs) have garnered extensive attention in clinical practice and have become a crucial treatment option for various malignancies, including non-small cell lung cancer (NSCLC), melanoma, head and neck squamous cell carcinoma, hepatocellular carcinoma, and other types of cancers (1–4). In a healthy body, the immune checkpoint pathway serves as a critical regulatory mechanism to prevent immune-mediated damage. However, this pathway can also be exploited by cancer cells to evade immune surveillance. Common targets for ICIs include cytotoxic T lymphocyte-associated protein-4 (CTLA-4), programmed cell death protein-1 (PD-1) and programmed death ligand-1 (PD-L1) (5).

CTLA-4 (cluster of differentiation 152, CD152), as a competitive inhibitor of CD28, inhibits the immune activity of CD8^+^ T cells by blocking the co-stimulatory signals mediated by the interaction between CD28 and B7-1 (CD80) or B7-2 (CD86) on antigen-presenting cells (APCs) (6). PD-L1 (B7-H1 or CD274), expressed on the surface of tumor cells as a ligand for PD-1, can inhibit T cell activation and negatively regulate the adaptive immune response, thereby enabling tumors to evade immune surveillance (7). Antibodies directed against PD-1/PD-L1 can inhibit immune evasion and augment adaptive immune responses, leading to more effective tumor cell elimination (7, 8). Currently, ICIs approved for clinical use encompass a range of agents, including pembrolizumab, toripalimab, avelumab, and atezolizumab, among others (9–12).

Tremelimumab (Imjudo) is a human IgG2 monoclonal antibody that specifically binds to CTLA-4, blocking its interaction with the ligands, and enhances T cell activation and responses against tumor cells. In October 2022, tremelimumab received approval in the United States for use in combination with PD-L1 inhibitor durvalumab (Imfinzi) to treat unresectable hepatocellular carcinoma in adults (13). In clinical practice, the innovative strategy of initiating treatment with a low-dose CTLA-4 inhibitor followed by maintenance therapy with a PD-L1 inhibitor, referred to as the STRIDE regimen (Single Tremelimumab Regular Interval Durvalumab), provides synergistic therapeutic effects while substantially reducing immune-related toxicities, thereby demonstrating an improved safety profile (14). Ipilimumab (Yervoy), an additional CTLA-4 inhibitor, has also been evaluated in combination with PD-1 inhibitor nivolumab (Opdivo) as a first-line treatment option for metastatic NSCLC (15). It is widely recognized as the most classic treatment regimen within the combination therapies involving PD-1 inhibitors and CTLA-4 inhibitors (16).

The concern is that the use of these medications is not exclusively associated with a favorable prognosis, even though they are considered safer and more conventional treatment options. Single immune checkpoint inhibitors (sICIs) are frequently reported to cause adverse events (AEs) such as rash, jaundice, hypothyroidism, pneumonitis, neutropenia, and in some cases, necessitate medication discontinuation (17–21). We can also identified reports of AEs associated with dual immune checkpoint inhibitors (ipilimumab plus nivolumab or tremelimumab plus durvalumab, dICIs), including nausea, fatigue, pneumonia, neutropenia, leukopenia, and instances of treatment discontinuation (13, 15). However, limited specific comparisons of these AEs have been conducted between sICIs and dICIs.

Although dICIs are currently approved for various cancer indications and have demonstrated superior efficacy compared to sICI therapy, their safety profile and limitations warrant careful consideration (22). Given the inherent limitations of clinical trials, the US Food and Drug Administration Adverse Event Reporting System (FAERS) database serves as a spontaneous reporting system for pharmacovigilance to assess the safety of suspected AEs. To gain a deeper understanding of AEs associated with dICI, refine knowledge of its toxicity spectrum, and provide valuable clinical information, we conducted an in-depth analysis of the FAERS database.

## Materials and methods

### Data source

The original data utilized for our data mining were sourced exclusively from the FAERS database available on the official FDA website. The raw ASCII data files were downloaded for subsequent data mining and statistical analysis. SAS 9.4 software, recommended by the FDA for FAERS database analysis, was employed for this purpose. It is important to note that the FAERS database contains self-reported data, which may include duplicate or withdrawn/deleted reports. In accordance with the FDA’s official guidance documents, we conducted data cleaning as follows: We selected the PRIMARYID, CASEID, and FDA_DT fields from the DEMO table, sorted them by CASEID, FDA_DT, and PRIMARYID, and retained the maximum values in sequence. Since the first quarter of 2019, each quarterly data package has included a list of deleted reports. After deduplication, we removed the deleted reports based on the CASEID provided in the deletion list. The latest version of the Medical Dictionary for Regulatory Activities (MedDRA 27.0), which includes system organ class (SOC) and preferred term (PT), was systematically utilized to encode the AE terminology within the FAERS database.

### Statistical analysis

For the analysis of baseline data, we employed Cramer’s V coefficient in conjunction with the p-value to evaluate both the strength and statistical significance of associations between categorical variables, particularly for those with larger sample sizes. A p-value more than 0.05 accompanied by a V value close to zero suggests a weak association between the variables. Such a weak association may arise by chance and is therefore considered to have limited reliability. And we employed a case-control study design, specifically a case versus non-case approach, utilizing a fourfold table for disproportionality analysis, as illustrated in **Table 1**, to investigate the AEs associated with the target medications. In the database, each patient (represented by an individual report) is associated with a unique “Primary Suspect (PS)” medication. When defining the target medication user population, only the patient’s PS medication is taken into account. If the PS medication recorded in the background database matches the target medication of the study, the patient is included in the target medication population; otherwise, the patient is classified into the other medication population.

**Table 1:** The fourfold table for disproportionality analysis.

Fourfold table	Target AE	All other AEs	Total
Target medication	*a*	*b*	*a*+*b*
All other medications	*c*	*d*	*c*+*d*
Total	*a*+*c*	*b*+*d*	*n*=*a+b+c+d*

The level of AEs is used for analysis. Background: AEs are observed in patients receiving monotherapy with tremelimumab, durvalumab, ipilimumab, or nivolumab, as well as in those receiving combination therapy with tremelimumab plus durvalumab or ipilimumab plus nivolumab.

**Figure 1:**
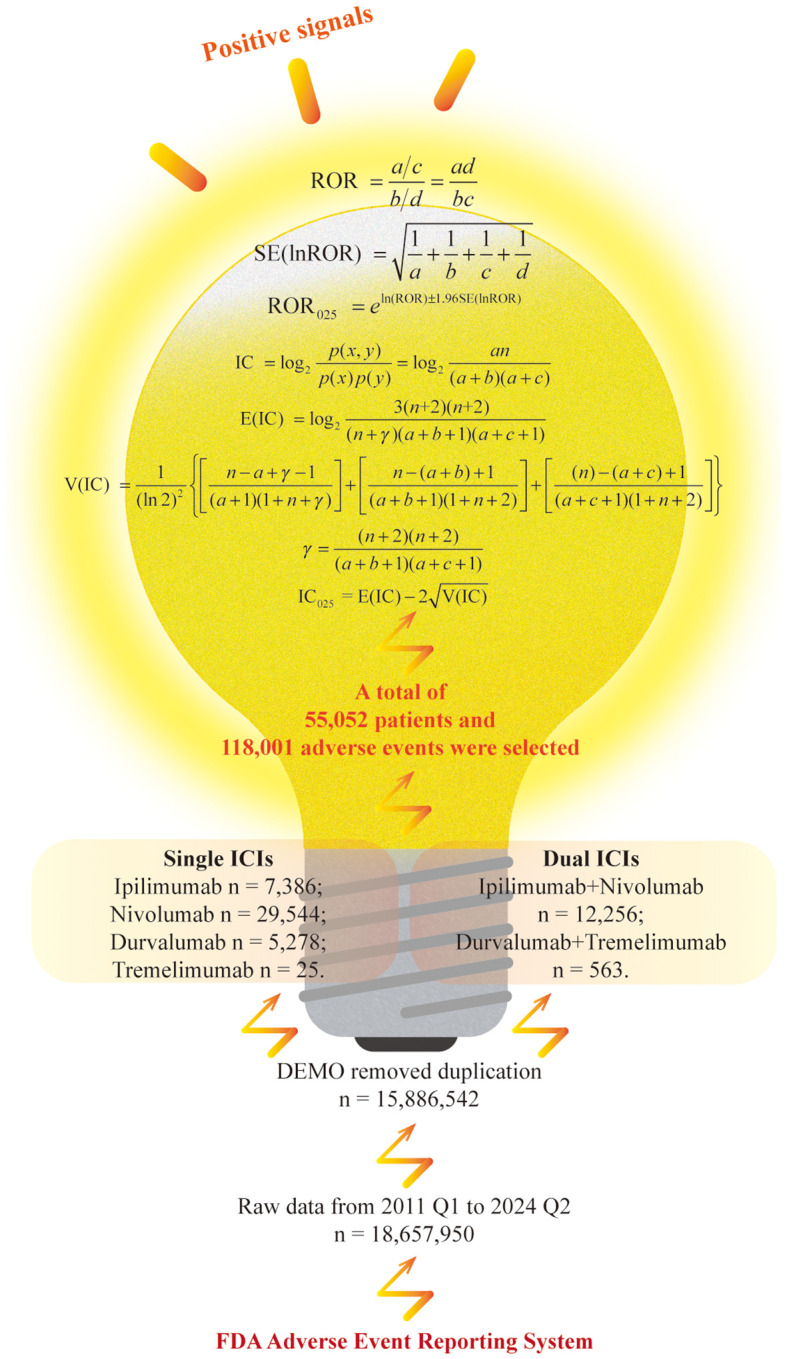
Flowchart for data filtering and analysis.

In this study, we employed widely recognized methods to detect adverse medication event signals. Specifically, positive signals were identified using the reporting odds ratio (ROR) and the Bayesian confidence propagation neural network (BCPNN) (23, 24). For the ROR method, a signal is triggered when the analysis data satisfies *a* ≥ 3 and 95% confidence interval (CI) lower limit of ROR (ROR_025_) > 1. For the BCPNN method, a signal is generated if 95% CI lower limit of the information component (IC_025_) > 0. As illustrated in **Figure 1**, a positive signal is produced when the AE included in the analysis meets the criteria for both methods.

ROR is a disproportionality analysis method based on the fourfold contingency table, characterized by high sensitivity in detecting typical AEs associated with commonly used medications. It considers the total number of reports and allows for adjustment of potential confounding factors (25, 26). And BCPNN is an advanced algorithm grounded in Bayesian theory, designed to evaluate the likelihood of a causal association between medications and AEs. This method demonstrates superior performance in handling sparse data compared to conventional approaches and tends to produce fewer false-positive signals. Furthermore, the Bayesian framework of BCPNN inherently accounts for reporting bias by incorporating prior distributions to mitigate spurious associations (27, 28). Therefore, this study employs both the ROR and BCPNN methods, which together can help minimize bias resulting from proportional imbalances and are widely recognized for their effectiveness in mining and analyzing similar pharmacovigilance databases such as FAERS (29, 30).

## Results

### Descriptive results

The data screening and analysis process is illustrated in **Figure 1**. A total of 54 quarters of ASCII packets were downloaded from the FAERS database, covering the period from the first quarter of 2011 to the second quarter of 2024, resulting in the acquisition of 18,657,950 records. After eliminating duplicate reports, 15,886,542 unique reports remained. Subsequently, we screened for reports where the target medication was listed as the PS medication, selecting a total of 118,001 AEs involving 55,052 individuals as the background dataset. Specifically, among sICIs, there were 7,386 reports for ipilimumab, 29,544 for nivolumab, 5,278 for durvalumab, and 25 for tremelimumab. For dICIs, 12,256 participants reported receiving ipilimumab combined with nivolumab, while 563 people received durvalumab and tremelimumab.

**Figure 2:**
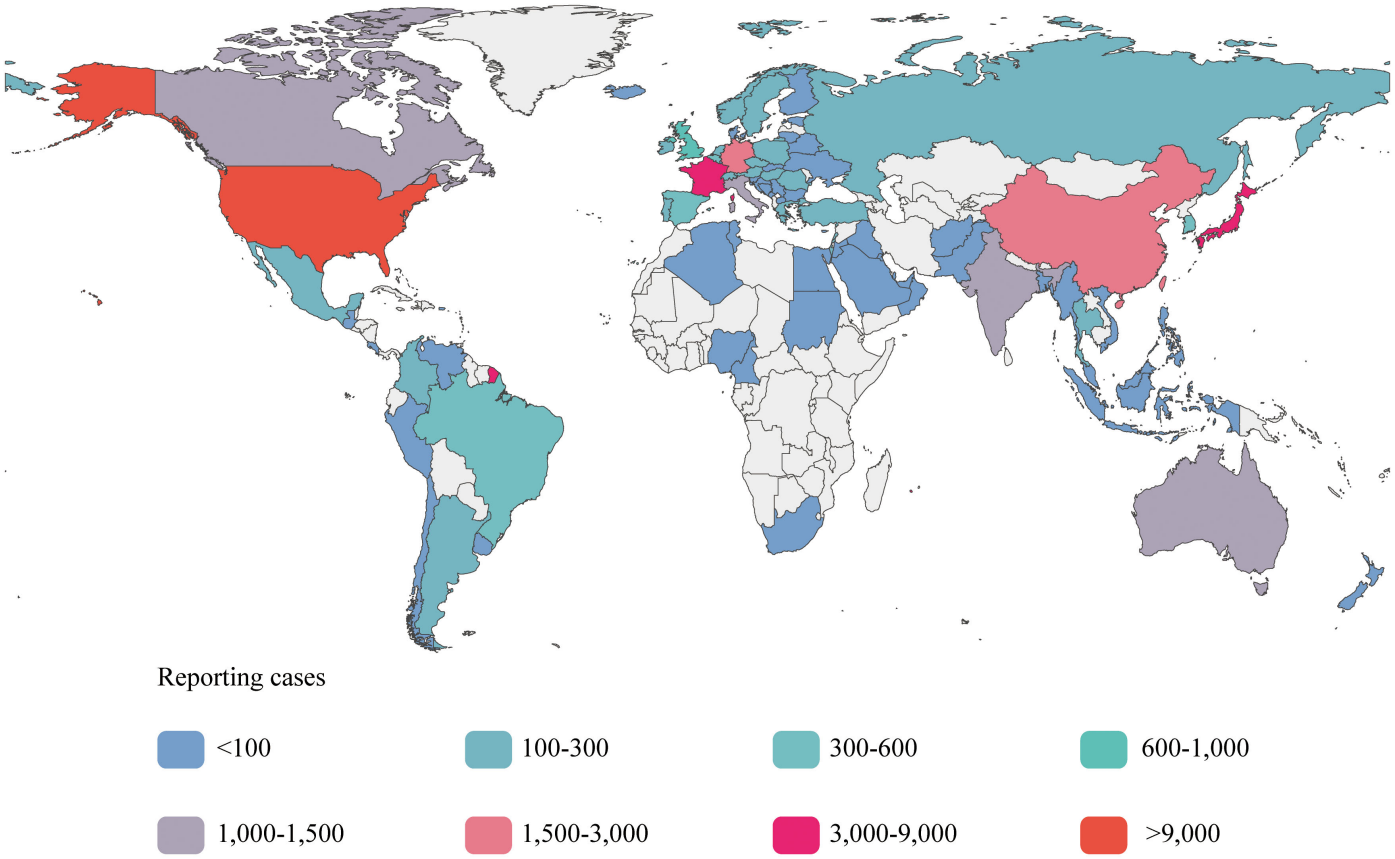
Reporting countries.

Among the six subgroups included in the study, patients who received ipilimumab, nivolumab, tremelimumab, or durvalumab as monotherapies were classified as sICIs. Patients who received combination therapies of ipilimumab plus nivolumab or tremelimumab plus durvalumab were classified into the dual immunotherapy group (DIG). We have presented the baseline data for the sICIs and DIG in **Table 2**, and provided detailed data pertaining to the six subgroups in **Additional File 1**. After excluding unspecified cases, 51.73% of patients were male, with 51.19% in the sICIs and 53.54% in the DIG, both proportions exceeding those of female patients. The highest frequency of AEs was observed in the age group of 45–64 years, accounting for 20.81% in the sICIs and 25.58% in the DIG. In the DIG, the majority of AE reports were submitted by physicians (44.71%), whereas in the sICIs, consumers (32.26%) and physicians (31.94%) were the primary reporters. Regarding the sources of the reports, **Figure 2** presents a choropleth map illustrating the countries contributing to the reports. The majority of the reports originate from the United States (25,788, 46.85%) and Japan (8,794, 15.98%), followed by France (4,031, 7.32%) and China (2,697, 4.90%). Notably, each of these four countries has contributed more than 2,000 reports, whereas the others have not. Serious reports in the FAERS database are categorized at the patient level rather than at the level of specific AEs. Reports that include outcome results are classified as serious (47,350, 86.01%), whereas those lacking outcome information are deemed non-serious (7,702, 13.99%).

**Table 2:** Baseline information of sICIs and DIG.

Indicator (%)	sICIs (N = 42,233)	DIG (N = 12,819)	Total
Gender Cramer’s V 0.036, p < 0.001			
Female	1,1721 (27.75)	3,691 (28.79)	15,412 (28.00)
Male	2,1617 (51.19)	6,863 (53.54)	28,480 (51.73)
Unspecified	8,895 (21.06)	2,265 (17.67)	11,160 (20.27)
Age Cramer’s V 0.114, p < 0.001			
<18	82 (0.19)	28 (0.22)	110 (0.20)
18-44	1,654 (3.92)	803 (6.26)	2,457 (4.46)
45-64	8,787 (20.81)	3,314 (25.85)	12,101 (21.98)
65-74	7,678 (18.18)	2,745 (21.41)	10,423 (18.93)
≥75	4,776 (11.31)	1,738 (13.56)	6,514 (11.83)
Unspecified	19,256 (45.59)	4,191 (32.69)	23,447 (42.59)
Age (quantitative)			
N (Missing)	22,977 (19,256)	8,628 (4,191)	31,605 (23,447)
Mean (SD)	64.14 (12.96)	63.10 (13.57)	63.86 (13.13)
Median (Q1, Q3)	66.00 (57.00, 73.00)	65.00 (55.00, 73.00)	66.00 (57.00, 73.00)
Min, Max	0.00, 120.00	0.00, 100.00	0.00, 120.00
Reporting year Cramer’s V 0.296, p < 0.001			
2011	263 (0.62)		263 (0.48)
2012	839 (1.99)		839 (1.52)
2013	649 (1.54)	3 (0.02)	652 (1.18)
2014	972 (2.30)	9 (0.07)	981 (1.78)
2015	1,802 (4.27)	113 (0.88)	1,915 (3.48)
2016	4,648 (11.01)	381 (2.97)	5,029 (9.13)
2017	5,788 (13.70)	741 (5.78)	6,529 (11.86)
2018	4,970 (11.77)	1,068 (8.33)	6,038 (10.97)
2019	5,368 (12.71)	1,671 (13.04)	7,039 (12.79)
2020	4,500 (10.66)	1,683 (13.13)	6,183 (11.23)
2021	4,256 (10.08)	1,754 (13.68)	6,010 (10.92)
2022	3,745 (8.87)	2,056 (16.04)	5,801 (10.54)
2023	2,801 (6.63)	1,889 (14.74)	4,690 (8.52)
2024	1,632 (3.86)	1,451 (11.32)	3,083 (5.60)
Reporter Cramer’s V 0.191, p < 0.001			
Consumer	13,625 (32.26)	1,987 (15.50)	15,612 (28.36)
Lawyer	20 (0.05)	6 (0.05)	26 (0.05)
Unspecified	816 (1.93)	29 (0.23)	845 (1.53)
Other health-professional	6,856 (16.23)	1,756 (13.70)	8,612 (15.64)
Pharmacist	7,425 (17.58)	3,310 (25.82)	10,735 (19.50)
Physician	13,491 (31.94)	5,731 (44.71)	19,222 (34.92)
Serious report Cramer’s V 0.079, p < 0.001				
Serious	35,686 (84.50)	11,664 (90.99)	47,350 (86.01)
Non-Serious	6,547 (15.50)	1,155 (9.01)	7,702 (13.99)
Outcome: Life-Threatening Cramer’s V 0.056, p < 0.001				
Yes	1,559 (3.69)	820 (6.40)	2,379 (4.32)
No	40,674 (96.31)	11,999 (93.60)	52,673 (95.68)
Outcome: Hospitalization - Initial or Prolonged Cramer’s V 0.132, p < 0.001				
Yes	10,091 (23.89)	4,845 (37.80)	14,936 (27.13)
No	32,142 (76.11)	7,974 (62.20)	40,116 (72.87)
Outcome: Disability Cramer’s V 0.004, p = 0.411				
Yes	509 (1.21)	143 (1.12)	652 (1.18)
No	41,724 (98.79)	12,676 (98.88)	54,400 (98.82)
Outcome: Death Cramer’s V 0.051, p < 0.001				
Yes	12,985 (30.75)	3,238 (25.26)	16,223 (29.47)
No	29,248 (69.25)	9,581 (74.74)	38,829 (70.53)
Outcome: Congenital Anomaly Cramer’s V 0.002, p = 0.566				
Yes	15 (0.04)	6 (0.05)	21 (0.04)
No	42,218 (99.96)	12,813 (99.95)	55,031 (99.96)
Outcome: Required Intervention to Prevent Permanent Impairment Cramer’s V 0, p = 0.912				
Yes	41 (0.10)	12 (0.09)	53 (0.10)
No	42,192 (99.90)	12,807 (99.91)	54,999 (99.90)
Outcome: Other Serious Report Cramer’s V 0.078, p < 0.001				
Yes	29,897 (70.79)	10,135 (79.06)	40,032 (72.72)
No	12,336 (29.21)	2,684 (20.94)	15,020 (27.28)
Onset Time Cramer’s V 0.089, p < 0.001				
< 30 days	3,628 (8.59)	1,686 (13.15)	5,314 (9.65)
31–60 days	1,604 (3.80)	744 (5.80)	2,348 (4.27)
61–90 days	1,021 (2.42)	430 (3.35)	1,451 (2.64)
91–120 days	685 (1.62)	228 (1.78)	913 (1.66)
121–150 days	435 (1.03)	142 (1.11)	577 (1.05)
151–180 days	343 (0.81)	79 (0.62)	422 (0.77)
181–360 days	1,051 (2.49)	224 (1.75)	1,275 (2.32)
> 360 days	692 (1.64)	149 (1.16)	841 (1.53)
Missing	32,774 (77.60)	9,137 (71.28)	41,911 (76.13)
Onset Time (quantitative)				
N (Missing)	9,459 (32,774)	3,682 (9,137)	13,141 (41,911)
Mean (SD)	123.72 (255.85)	77.11 (144.63)	110.66 (231.12)
Median (Q1, Q3)	50.00 (16.00, 131.00)	36.00 (14.00, 82.00)	44.00 (15.00, 114.00)
Min, Max	0.00, 7,124.00	0.00, 4,378.00	0.00, 7,124.00

DIG, dual immunotherapy group, ipilimumab+nivolumab and tremelimumab+durvalumab; sICIs, single ipilimumab, nivolumab, tremelimumab, or durvalumab; SD, standard deviation; Q1, lower quartile; Q3, upper quartile. The patient level is utilized for statistical description. The onset time recorded in the database refers to the date when a patient first experiences any AE, not a particular AE. When calculating onset time, values less than 0 days are included after imputing missing data.

**Figure 3:**
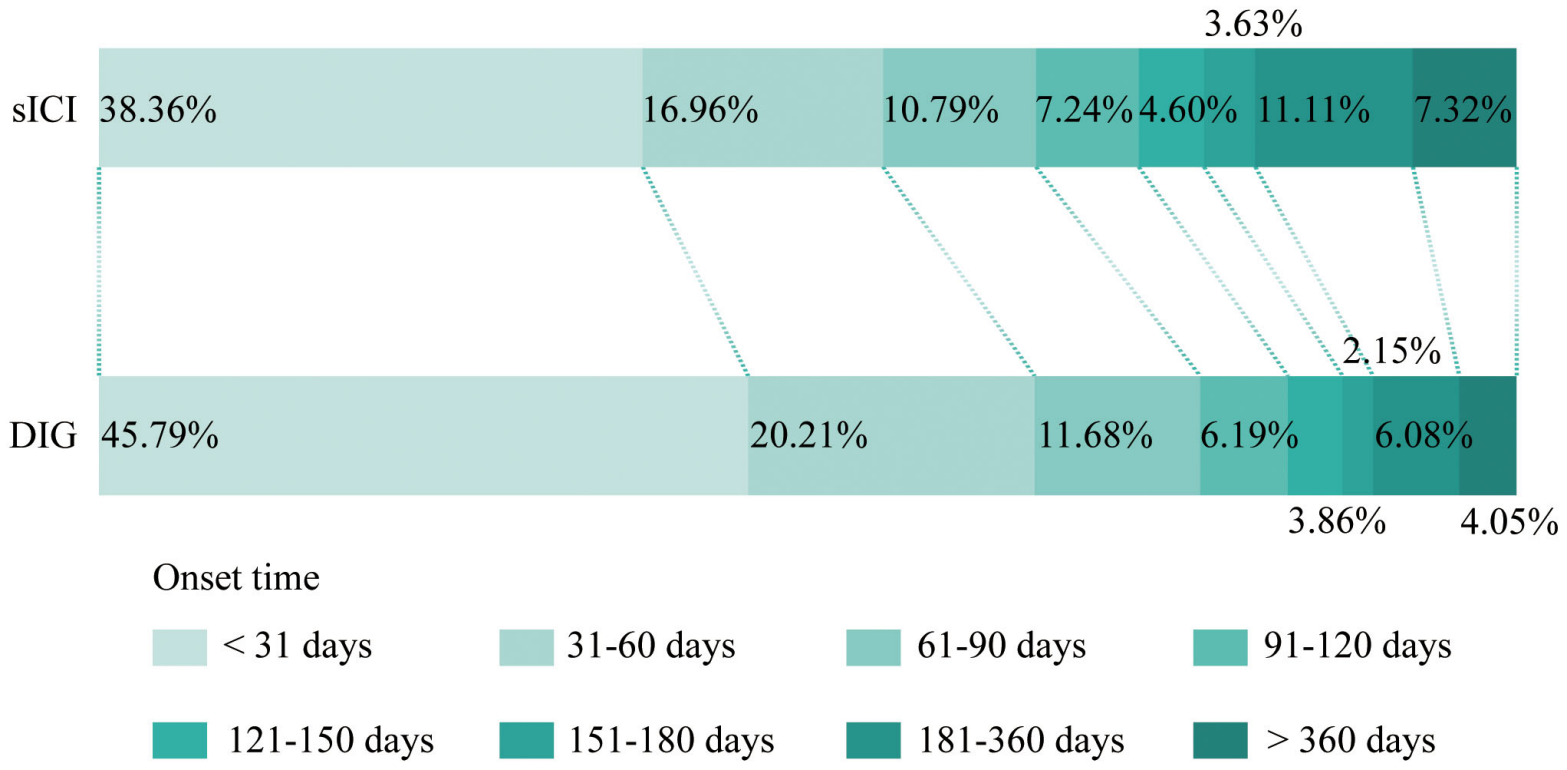
Onset time of sICI and DIG.

Onset time refers to the duration from the initiation of medication until the occurrence of AEs. As shown in **Figure 3**, after excluding reports with missing or abnormal onset times, DIG exhibits an earlier first AE compared to sICIs. For this processed dataset, we focus primarily on the median values (**Table 2**). The median onset time for DIG is 36 days (interquartile range 14–82 days), which is notably shorter than that of sICIs at 50 days (interquartile range 16–131 days). It is important to note that a substantial proportion of onset time data is missing (76.13%), which may potentially introduce significant bias into the interpretation and reporting of onset time results.

### Positive signals of DIG at SOC level

**Figure 4:**
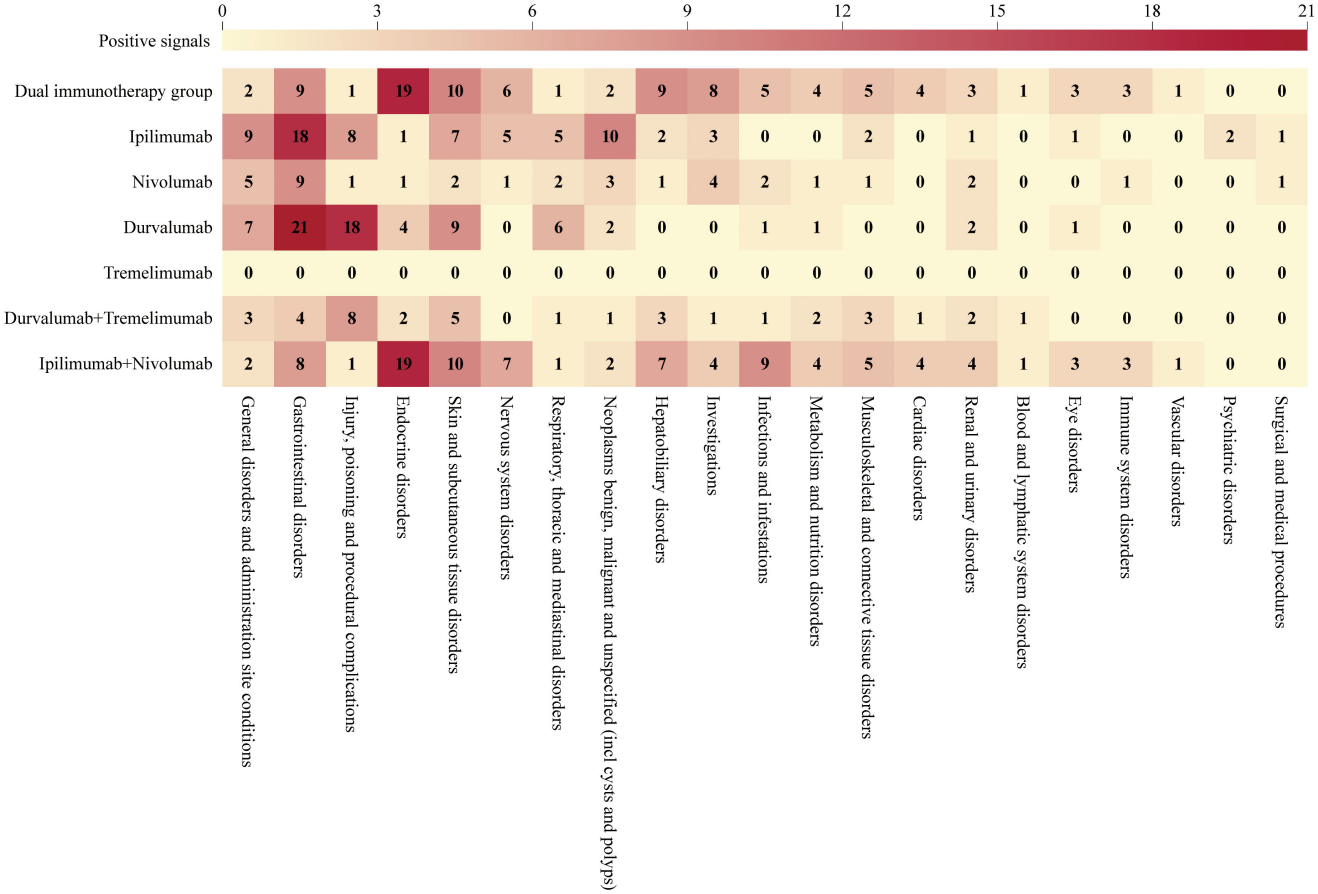
Positive signals at SOC level.

We analyzed the target AEs using two algorithms, ROR and BCPNN. An AE positive signal was generated only when both algorithms met their respective signal generation conditions simultaneously. As illustrated in **Figure 4**, at the SOC level, DIG (primarily derived from ipilimumab plus nivolumab) demonstrated significant AEs in detecting endocrine disorders (SOC: 10014698), with 19 distinct types of positive signals identified. In contrast, sICIs exhibited fewer types of positive signals. Moreover, DIG detected a higher number of AE types across multiple SOCs, including skin and subcutaneous tissue disorders (10 PT types, SOC: 10040785), nervous system disorders (6 PT types, SOC: 10029205), hepatobiliary disorders (9 PT types, SOC: 10019805), investigations (8 PT types, SOC: 10022891), infections and infestations (5 PT types, SOC: 10021881), metabolism and nutrition disorders (4 PT types, SOC: 10027433), musculoskeletal and connective tissue disorders (5 PT types, SOC: 10028395), cardiac disorders (4 PT types, SOC: 10007541), renal and urinary disorders (3 PT types, SOC: 10038359), blood and lymphatic system disorders (1 PT type, SOC: 10005329), eye disorders (3 PT types, SOC: 10015919), immune system disorders (3 PT types, SOC: 10021428), and vascular disorders (1 PT type, SOC: 10047065) compared to sICIs.

Tremelimumab exhibited a very low number of positive signals, likely attributable to the limited number of reports. Besides tremelimumab, fewer types of positive signals were observed in several other categories: general disorders and administration site conditions (2 PT types, SOC: 10018065), injury, poisoning and procedural complications (1 PT type, SOC: 10022117), respiratory, thoracic and mediastinal disorders (1 PT type, SOC: 10038738), as well as neoplasms benign, malignant and unspecified (incl cysts and polyps) (2 PT types, SOC: 10029104).

### Positive signals of DIG at PT level

**Figure 5:**
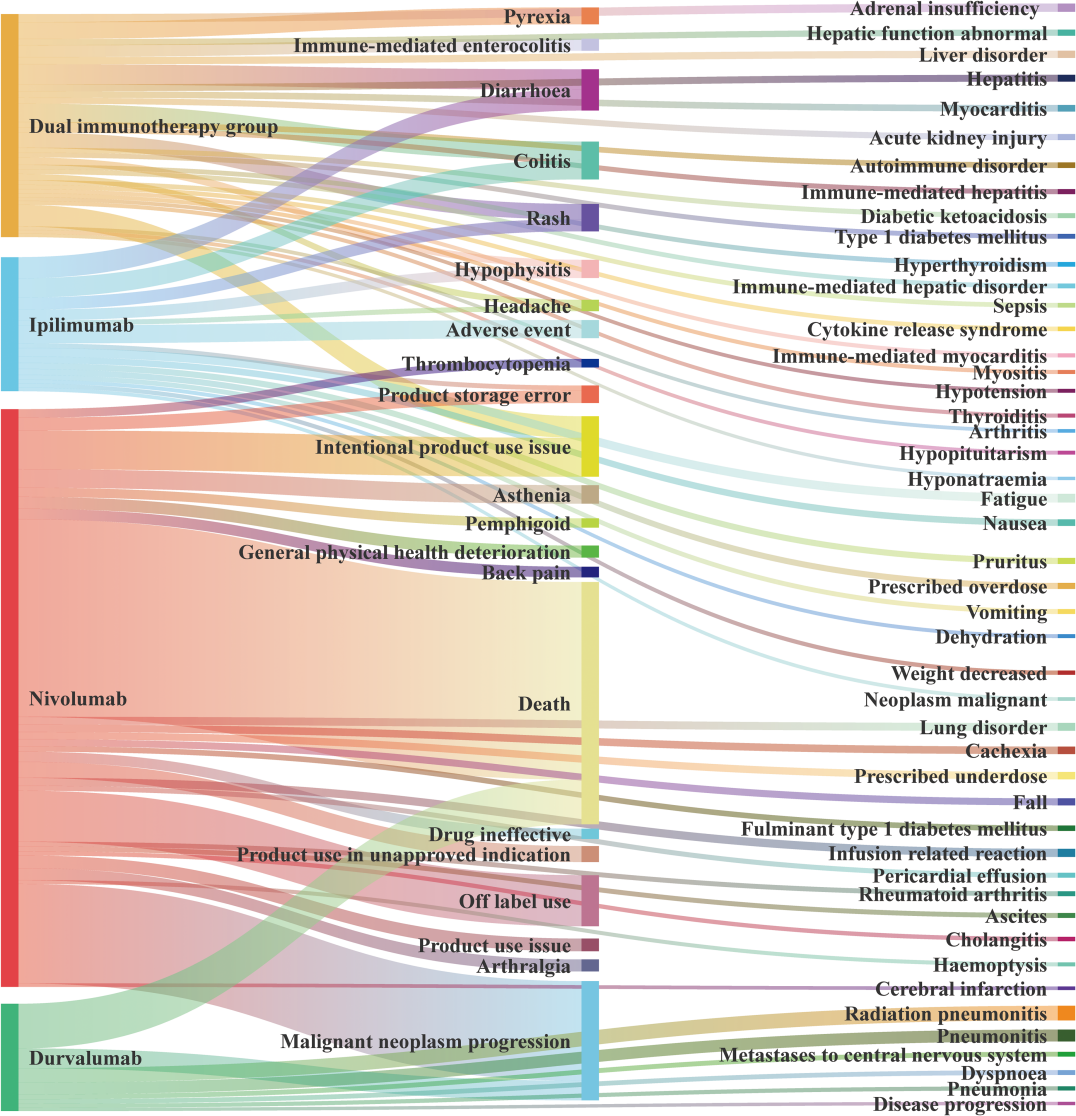
Sources of positive signals for AE types.

We present the AEs with *a* > 100, summarized in **Figure 5** according to the source of the positive signal. In DIG, the most frequently reported AE was intentional product use issue (*a* = 679), followed by diarrhea (*a* = 633), colitis (*a* = 581), pyrexia (*a* = 530), and others. In sICI, the top three AEs associated with ipilimumab were diarrhea (*a* = 665), colitis (*a* = 607), and adverse event (*a* = 566, PT: 10060933). For nivolumab, the leading AEs were death (*a* = 6,216), malignant neoplasm progression (*a* = 3,129), and off-label use (*a* = 1,605). Durvalumab was associated with death (*a* = 1,405), malignant neoplasm progression (*a* = 619), and radiation pneumonitis (*a* = 456).

**Figure 6:**
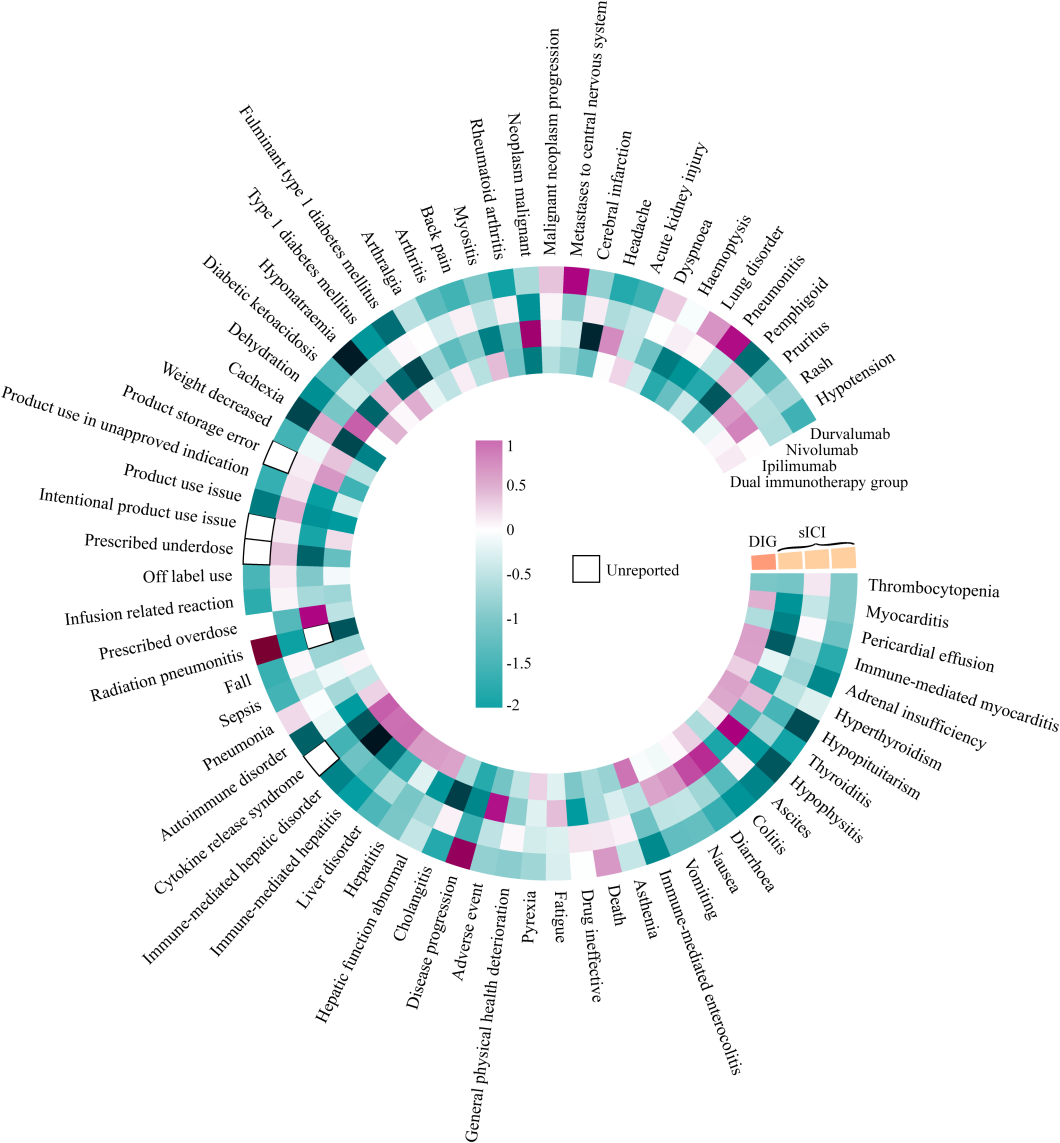
Heatmap of IC_025_ signal at PT level.

The data were analyzed using two methods, ROR and BCPNN, with detailed results provided in **Additional File 2**. In **Figure 6**, we summarize the IC_025_ of positive signals with *a* > 100 obtained by the BCPNN method for DIG and sICIs (including non-positive signals). DIG exhibited higher positive signal intensity compared to sICIs across the following PTs: myocarditis (ROR 2.221, IC_025_ 0.486, PT: 10028606), immune-mediated myocarditis (ROR 2.922, IC_025_ 0.610, PT: 10082606), adrenal insufficiency (ROR 2.503, IC_025_ 0.602, PT: 10001367), hyperthyroidism (ROR 1.872, IC_025_ 0.305, PT: 10020850), thyroiditis (ROR 2.669, IC_025_ 0.546, PT: 10043778), immune-mediated enterocolitis (ROR 3.948, IC_025_ 0.937, PT: 10078961), pyrexia (ROR 1.570, IC_025_ 0.290, PT: 10037660), hepatic function abnormality (ROR 2.582, IC_025_ 0.591, PT: 10019670), hepatitis (ROR 2.705, IC_025_ 0.637, PT: 10019717), liver disorder (ROR 2.718, IC_025_ 0.646, PT: 10024670), immune-mediated hepatitis (ROR 5.504, IC_025_ 0.994, PT: 10078962), immune-mediated liver disorder (ROR 5.322, IC_025_ 0.966, PT: 10083521), cytokine release syndrome (ROR 7.650, IC_025_ 1.103, PT: 10052015), autoimmune diseases (ROR 1.754, IC_025_ 0.275, PT: 10061664), sepsis (ROR 1.414, IC_025_ 0.062, PT: 10040047), diabetic ketoacidosis (ROR 2.294, IC_025_ 0.472, PT: 10012671), type 1 diabetes mellitus (ROR 2.421, IC_025_ 0.508, PT: 10067584), arthritis (ROR 1.562, IC_025_ 0.113, PT: 10003246), myositis (ROR 2.204, IC_025_ 0.412, PT: 10028653), and acute kidney injury (ROR 1.708, IC_025_ 0.264, PT: 10069339). In contrast, the occurrence of death (PT: 10011906), product storage error (PT: 10079843), malignant neoplasm progression (PT: 10051398), and lung disorder (PT: 10025082) is lower in DIG compared to sICIs. The relevant ROR analysis results (only DIG ROR_025_ > 1) are presented in **Table 3**.

**Table 3:** AEs signal strength of DIG and sICI at the level of PTs (ROR only).

System organ classes and preferred terms	DIG ROR	DIG ROR_025_	Ipilimumab ROR	Ipilimumab ROR_025_	Nivolumab ROR	Nivolumab ROR_025_	Durvalumab ROR	Durvalumab ROR_025_
Cardiac disorders								
Myocarditis	2.221	1.843	0.263	0.160	0.748	0.621	0.728	0.493
Immune-mediated myocarditis	2.922	2.271	0.119	0.044	0.661	0.513	0.488	0.259
Endocrine disorders								
Adrenal insufficiency	2.503	2.111	1.138	0.888	0.511	0.428	0.240	0.132
Hyperthyroidism	1.872	1.515	0.403	0.254	0.713	0.578	1.207	0.847
Hypopituitarism	2.913	2.241	2.111	1.544	0.292	0.215	0.102	0.025
Thyroiditis	2.669	2.080	0.559	0.346	0.587	0.455	0.428	0.220
Hypophysitis	1.450	1.236	7.612	6.532	0.136	0.108	0.103	0.046
Gastrointestinal disorders								
Colitis	1.575	1.421	4.583	4.135	0.262	0.233	0.239	0.169
Diarrhea	1.193	1.086	3.491	3.178	0.459	0.419	0.376	0.294
Vomiting	1.192	1.012	2.019	1.683	0.673	0.578	0.557	0.391
Immune-mediated enterocolitis	3.948	3.369	0.884	0.689	0.345	0.290	0.237	0.137
General disorders and administration site conditions								
Pyrexia	1.570	1.410	0.988	0.843	0.742	0.668	0.725	0.583
Hepatobiliary disorders								
Hepatic function abnormal	2.582	2.120	0.281	0.168	0.578	0.472	1.035	0.725
Hepatitis	2.705	2.239	1.134	0.861	0.396	0.322	0.700	0.467
Liver disorder	2.718	2.258	0.437	0.294	0.593	0.491	0.529	0.337
Immune-mediated hepatitis	5.504	4.188	0.189	0.084	0.333	0.249	0.404	0.200
Immune-mediated hepatic disorder	5.322	4.021	0.033	0.005	0.404	0.304	0.266	0.109
Cytokine release syndrome	7.650	5.520	0.116	0.037	0.293	0.210		
Autoimmune disorder	1.754	1.447	0.306	0.191	1.122	0.929	0.131	0.054
Infections and infestations								
Sepsis	1.414	1.155	1.239	0.946	0.787	0.649	0.509	0.317
Injury, poisoning and procedural complications								
Intentional product use issue	1.422	1.295	0.290	0.231	1.461	1.334		
Metabolism and nutrition disorders								
Dehydration	1.349	1.082	3.536	2.834	0.409	0.327	0.293	0.151
Diabetic ketoacidosis	2.294	1.849	0.130	0.058	0.817	0.659	0.572	0.346
Hyponatraemia	1.475	1.161	2.025	1.537	0.661	0.524	0.039	0.005
Type 1 diabetes mellitus	2.421	1.940	0.138	0.062	0.833	0.668	0.333	0.172
Arthritis	1.562	1.239	0.574	0.375	0.929	0.743	0.621	0.375
Myositis	2.204	1.742	0.209	0.103	0.752	0.595	0.779	0.483
Nervous system disorders								
Headache	1.284	1.084	2.403	2.002	0.584	0.497	0.399	0.258
Renal and urinary disorders								
Acute kidney injury	1.708	1.422	0.954	0.723	0.749	0.626	0.490	0.313
Skin and subcutaneous tissue disorders								
Rash	1.230	1.104	2.357	2.096	0.552	0.498	0.711	0.575
Vascular disorders								
Hypotension	1.579	1.256	1.448	1.077	0.648	0.518	0.529	0.309

ROR, reporting odds ratio; BCPNN, Bayesian confidence propagation neural network; DIG, dual immunotherapy group; sICI, single immune checkpoint inhibitor; ROR_025_, 95% CI lower limit of ROR; PT, preferred term. Only medication groups with *a* > 100 and ROR_025_ > 1, positive ROR signals are displayed. Information related to IC_025_ will be presented in the figures and supplementary materials. The data shown in the table includes PTs that fail to meet both the ROR and BCPNN positive signal criteria.

### Positive signals of Durvalumab + Tremelimumab and Durvalumab at PT level

Due to the promotion of the STRIDE regimen, the combination of Durvalumab and Tremelimumab has been more widely adopted in clinical practice compared to Tremelimumab monotherapy, largely due to its improved safety profile. Comparing the safety signals between the Durvalumab + Tremelimumab group and the Durvalumab monotherapy group can more clearly illustrate the differences in their safety profiles. We present the AEs with positive and strongly positive IC signal intensities in **Additional File 3**. The results indicate that the use of Durvalumab alone is strongly associated with AEs such as radiation pneumonitis (PT: 10037765), recurrence of non-small cell lung cancer (PT: 10029515), and disease progression (PT: 10061818), whereas these associations are not observed in the Durvalumab + Tremelimumab group. However, it should be noted that the use of this dICI regimen is strongly linked to AEs including cytokine release syndrome (PT: 10052015) and ruptured liver carcinoma (PT: 10050842).

## Discussion

Previous studies have reported that the most frequently observed AEs associated with dICIs were predominantly dermatological (34% for any grade and 4.2% for grade 3 or higher), endocrine (23.8% for any grade and 4.2% for grade 3 or higher), gastrointestinal (18.2% for any grade and 2.4% for grade 3 or higher), and hepatic (15.8% for any grade and 8.2% for grade 3 or higher) (22). Through deep mining and signal analysis of the FAERS database, we identified a higher incidence of immune-related adverse events (irAEs) associated with DIG, including immune-mediated myocarditis, immune-mediated enterocolitis, immune-mediated hepatitis, and immune-mediated liver disorders. Moreover, the pronounced signal intensity associated with cytokine release syndrome (CRS) merits thorough investigation. For pyrexia, sepsis, arthritis, and other AEs with low signal intensity, this does not imply that the risks and challenges they pose in clinical practice can be overlooked.

Khoja et al. summarized in their review that the risk of irAEs varies depending on the type of ICI. Specifically, PD-1/PD-L1 inhibitors have a lower incidence of irAEs compared to CTLA-4 inhibitors, and the gastrointestinal tract, liver, skin, and thyroid are the most commonly affected organs (31). Furthermore, the toxicity associated with CTLA-4 inhibitors exhibits a dose-dependent relationship (32–34). The administration of dICI can enhance CTLA-4-induced expansion of CD4^+^/CD8^+^ T cells and reduce regulatory T cells (Tregs) in tumors, this phenomenon is not observed with sICI alone (35).

In all irAEs reporting positive signals, immune-mediated myotoxicit is a rare but potentially fatal complication (36, 37). Current understanding of immune-mediated myocarditis pathogenesis includes the absence of central tolerance due to the lack of specific cardiac antigen expression in thymic epithelial cells, impaired peripheral tolerance, activation of autoreactive T cells, interaction between T cells and cardiac APCs to recognize cardiac peptides, and a positive feedback loop involving cytokines and effector T cells that leads to cardiac tissue infiltration (38). The current diagnostic criteria for ICI-related myocarditis encompass elevated serum troponin and natriuretic peptide levels, prolonged PR intervals on electrocardiography, atrioventricular block, ventricular arrhythmias, ST-segment depression, or T-wave inversion. Additionally, echocardiography and cardiac magnetic resonance imaging can provide supportive evidence of myocardial injury. Endomyocardial biopsy serves as the gold standard for diagnosing myocarditis when necessary. Upon diagnosis of ICI-related myocarditis, ICIs should be discontinued even in cases of mild toxicity. High-dose glucocorticoids are recommended for a minimum duration of 4 to 6 weeks, with continuous monitoring of troponin levels. For patients who exhibit an inadequate response to glucocorticoids, other immunomodulatory agents and conventional cardiac therapies may be considered (39).

Compared with sICI, dICI was associated with a significantly higher incidence of immune-mediated enterocolitis (OR 3.53, 95% CI 3.4-4.4) (40). Firstly, the administration of dICI can disrupt the balance of the intestinal microbiota, leading to the activation of T helper 1 and 17 cells, which subsequently results in inflammation of the intestinal mucosa (41, 42). Secondly, patients with immune-mediated enterocolitis exhibited significantly higher levels of CD4^+^ T cells and lower levels of Tregs compared to those without colitis, thereby markedly reducing the colitis-free interval (43). Fecal microbiota transplantation has been proposed as a therapeutic approach to alleviate inflammation in patients with ICI-related enteritis who are refractory to immunosuppressive therapy (44). Following fecal transplantation, the gut microbiota of recipients becomes more similar to that of donors, characterized by an expansion of certain bacteria such as *Akkermansia* and *Bifidobacterium*, which have been associated with reduced inflammation (45). Reducing the use of antibiotics that target anaerobic bacteria when necessary has been shown to mitigate the recurrence of ICI-associated enteritis and improve patient survival (45).

The mechanism of immune-related hepatotoxicity encompasses multiple facets. The disruption of immune tolerance leads to aberrant expression of major histocompatibility complex molecules, co-stimulatory molecules, and anti-inflammatory cytokines in the liver, thereby mediating hepatic injury (46). Concurrently, dICI administration induces tumor cell lysis, resulting in epitope spreading and subsequent uptake by APCs (47). Furthermore, CTLA-4 inhibitors enhance the diversity of CD4^+^ and CD8^+^ T cells while diminishing the suppressive function of Tregs, as evidenced by biopsy findings from patients experiencing immune-related hepatotoxicity (48–50). CD4^+^/CD8^+^ T cells play a crucial role in the digestive system-related AEs mentioned above. Kawashima et al. proposed that CD4^+^ T cells are essential for supporting both B cells and CD8^+^ T cells (51). Moreover, CD4^+^/CD8^+^ T cells exhibit a significant association with CTLA-4 inhibitors in dICI (35). The application of CTLA-4 inhibitors may serve as a critical factor contributing to dICI-related AEs in clinical settings, which requires further investigation to better understand the balance between PD-1/PD-L1 inhibitors and CTLA-4 inhibitors in clinical practice.

The reporting of ICI-related CRS is still in its early stages, and awareness of CRS as an irAE remains insufficient, despite the compelling positive findings from this study (52–54). Currently, it is widely recognized that the mechanism underlying ICI-induced CRS involves excessive activation of the immune system. Specifically, the “dual release of immune brakes” mechanism associated with dICI serves as the foundational trigger for CRS. Activation of innate immune cells leads to the release of cytokines, which subsequently stimulate T and B cells within the adaptive immune system. Prolonged hyperactivation disrupts the normal negative feedback mechanisms that typically constrain immune-mediated damage, resulting in a positive feedback loop that amplifies the “cytokine cascade”. This self-perpetuating cycle promotes the accumulation of immune cells, sustained cytokine release, and further recruitment and activation of innate immune cells, thereby destabilizing the hot tumor microenvironment and leading to systemic immune cell infiltration, which ultimately precipitates CRS (55, 56). The specific mechanism of CRS induced by tumor treatment varies depending on the type of therapy. In this study, the combination of Durvalumab and Tremelimumab (ROR 15.811, IC_025_ 2.55, FDR-adjusted p < 0.001) demonstrated a stronger association with the occurrence of CRS compared to the combination of Ipilimumab and Nivolumab (ROR 4.448, IC_025_ 0.861, FDR-adjusted p < 0.001). This suggests that clinicians should remain vigilant regarding the potential development of CRS associated with dICIs in clinical practice. Further clinical trials and basic research are warranted to validate these findings and elucidate the underlying mechanisms. Notably, chimeric antigen receptor-T cell therapy has a higher incidence of CRS compared to ICI. However, this should not overshadow the fact that ICI also poses a significant risk of CRS (55, 57).

### Strength and limitation

We conducted an extensive analysis of the FAERS database to investigate the real-world toxicity profile of dICI, representing the most comprehensive evaluation of AE reports for dICI since its clinical introduction. This study aims to provide detailed insights into AE occurrences for clinical practice and establish a foundation for risk assessment in clinical decision-making. However, several limitations must be acknowledged. The FAERS database relies on voluntary reporting, where reporters may not possess specialized medical knowledge, and there is no requirement to provide evidence of a causal relationship between a drug and a specific AE. Furthermore, the incidence rate cannot be accurately determined based on these reports. AEs may be associated with the underlying medical condition being treated, concomitant medications, or other external factors. It should be noted that these reports only represent the observations and opinions of the individuals who submitted them. Moreover, the US Food and Drug Administration cannot capture all adverse event or medication error reports, and the quantity and quality of submitted reports are influenced by various factors.

## Conclusion

We utilized two algorithms, ROR and BCPNN, to comprehensively analyze the FAERS database. Our findings indicate that the AEs associated with dICI predominantly originate from irAEs, including myotoxicity, endocrine toxicity, and hepatotoxicity. Notably, CRS, a strongly positive signal and rarely reported AE, warrants particular attention in clinical decision-making.

## Data Availability

The original contributions presented in the study are included in the article/**Supplementary Material**. Further inquiries can be directed to the corresponding author.

## Supplementary material

Additional File 1 Baseline information of all medications. Description of data: Presentation of baseline data for 6 medication groups.

Additional File 2 AEs signal strength of all medications at the level of PTs (*a* > 100). Description of data: A demonstration of the intensity of medication-related AEs at the PT level for cases exceeding 100.

Additional File 3 AEs signal strength of Durvalumab + Tremelimumab and Durvalumab at the level of PTs (IC_025_ > 1.5) Description of data: A demonstration of the intensity of medication-related AEs at the PT level for IC_025_ > 1.5.

## Glossary

AE: Adverse event
APC: Antigen-presenting cells
BCPNN: Bayesian confidence propagation neural network
CD: Cluster of differentiation
CI: Confidence interval
CRS: Cytokine release syndrome
dICI: Dual immune checkpoint inhibitor/Ipilimumab+Nivolumab or Tremelimumab+Durvalumab
DIG: Dual immunotherapy group/Ipilimumab+Nivolumab and Tremelimumab+Durvalumab
FAERS: The US Food and Drug Administration Adverse Event Reporting System
IC_025_: 95% CI lower limit of information component
ICI: Immune checkpoint inhibitor
irAE: Immune-related adverse event
MedDRA: Medical Dictionary for Regulatory Activities
NSCLC: Non-small cell lung cancer
PS: Primary suspect
PT: Preferred term
Q1: Lower quartile
Q3: Upper quartile
ROR: Reporting odds ratio
ROR_025_: 95% CI lower limit of ROR
SD: Standard deviation
sICI: Single immune checkpoint inhibitor
SOC: System organ class
Treg: Regulatory T cell.
